# Investigating the Association Between Central Sensitization and Breathing Pattern Disorders

**DOI:** 10.3390/biomedicines13081982

**Published:** 2025-08-15

**Authors:** Hyunmo Lim, Yongwook Lee, Yechan Cha, Juhee Hwang, Hyojung Han, Huijin Lee, Jaeho Yang, Woobin Jeong, Yujin Lim, Donggeun Lee, Hyunjoong Kim

**Affiliations:** 1 Musculoskeletal Disease Prevention Exercise Center, Samsung Electronics, 100, Hanamsandan 6beon-ro, Gwangju 62218, Republic of Korea; gwg04273@naver.com; 2 Department of Physical Therapy, Gwangju Health University, Bungmun-daero 419 beon-gil, Gwangju 62287, Republic of Korea; serein25@naver.com (Y.L.); ckdpcks65@gmail.com (Y.C.); hjh78952@naver.com (J.H.); hyosy0@naver.com (H.H.); heejin0840@naver.com (H.L.); didwogh1234@gmail.com (J.Y.); jeongwoobin0716@naver.com (W.J.); a95229252@gmail.com (Y.L.); ldg5877@gmail.com (D.L.); 3 Department of Senior Exercise Prescription, Gwangju Health University, Bungmun-daero 419 beon-gil, Gwangju 62287, Republic of Korea

**Keywords:** central sensitization, chronic pain, pain catastrophizing, respiratory mechanics

## Abstract

**Background/Objectives:** Central sensitization (CS) is identified as a cause of pain in various musculoskeletal diseases, and breathing pattern disorders (BPDs) are reported to be correlated with chronic pain. This study aimed to analyze the relationship between CS and BPDs through regression analysis. **Methods:** A cross-sectional study was designed according to the strengthening the reporting of observational studies in epidemiology (STROBE) guidelines. Forty participants with moderate to extreme CS (central sensitization inventory for Koreans; CSI-K ≥ 40) were enrolled, and their respiratory motion (manual assessment of respiratory motion; MARM), respiratory function (self-evaluation of breathing questionnaire; SEBQ), respiratory muscle strength (maximal inspiratory pressure; MIP, maximal expiratory pressure; MEP), pain intensity (numeric pain rating scale; NPRS), pain cognition (Korean version of pain catastrophizing scale; K-PCS), muscle tone and stiffness were measured. **Results:** Among participants with moderate to extreme CS, 82.5% showed BPDs and 42.5% reported severe pain intensity. Regression analysis revealed significant relationships between respiratory and pain variables. K-PCS demonstrated significant negative relationships with MARM area (β = −0.437, R^2^ = 0.191) and positive relationships with SEBQ (β = 0.528, R^2^ = 0.279). In the subgroup with BPDs, strong regression relationships were found between MARM area and NPRS usual pain (β = −0.486, R^2^ = 0.237) and K-PCS (β = −0.605, R^2^ = 0.366). Multiple regression analysis showed that MARM area and SEBQ together explained 41.2% of variance in pain catastrophizing. The comprehensive muscle stiffness prediction model using CSI-K, K-PCS, and muscle tone showed remarkably high explanatory power (R^2^ = 0.978). **Conclusions:** In individuals with moderate to extreme CS, respiratory dysfunction was prevalent and significantly predictable through regression models with pain intensity and pain cognition. These quantitative regression relationships between breathing mechanics, pain measures, and muscle properties provide clinical prediction tools and suggest the importance of assessing breathing patterns in CS management.

## 1. Introduction

Central sensitization (CS) represents a state of neuronal hyperexcitability in the spinal cord that develops following tissue damage, encompassing various physiological mechanisms associated with increased pain response and sensitivity [1,2]. Notably, CS can persist independently of peripheral nociceptor sensitization and is defined as nociplastic pain resulting from neuroplastic changes due to chemical, structural, and functional alterations in the central nervous system [3]. When CS enhances pain responsiveness in the central nervous system, it leads to dysregulation of pain control mechanisms, resulting in hyperactivation of nociceptor-mediated stimuli [4,5]. This mechanism typically manifests in symptoms such as widespread pain, hyperalgesia, and allodynia [6].

CS is recognized as a primary mechanism that perpetuates chronic pain conditions even after tissue healing has occurred [7]. Common chronic pain-related disorders including fibromyalgia, rheumatoid arthritis, chronic back pain, and temporomandibular disorder are characterized by decreased pain thresholds and persistent pain states [8,9]. These chronic pain conditions not only cause physical discomfort but also significantly impact psychosocial factors [10]. Common psychosocial manifestations include anxiety, depression, and insomnia, which are closely correlated with pain intensity [11]. However, the increased pain intensity alone cannot fully explain these phenomena, and the amplification of central nervous system responsiveness due to CS is considered a key contributing factor [1,5]. Particularly significant is the increase in stress hormone secretion, such as cortisol, which induces hemodynamic and physiological changes through activation of the hypothalamic–pituitary–adrenal (HPA) axis [12]. Consequently, diaphragmatic breathing has gained attention as a strategy for improving psychosocial factors, known for its effectiveness in modulating central nervous system responses and reducing stress [13,14]. One study revealed that fibromyalgia patients demonstrated inefficient breathing patterns utilizing only accessory respiratory muscles, along with insufficient prefrontal cortex activation during diaphragmatic breathing exercises [15].

Breathing patterns influence both consciousness and physical state, and breathing pattern disorders (BPDs) can lead to physiological, mechanical, and psychological disruptions [16]. While normal breathing patterns are achieved through appropriate diaphragmatic activity, dysfunctional breathing patterns are characterized by reduced diaphragmatic function. Chronic pain patients frequently exhibit thoracic breathing patterns, primarily associated with excessive upper chest segment movement, and prolonged inappropriate breathing can cause problems even without specific symptoms [17]. This results in flattening and excessive tension of the diaphragm, reduced mobility, and increased reliance on intercostal muscles and accessory respiratory muscles for ventilation [18]. The persistent use of such unsustainable functional breathing patterns is likely to lead to chronic musculoskeletal issues and pain [19].

Based on the understanding that chronic pain and disability are influenced by psychological and social factors beyond pathological changes, multidisciplinary interventions for chronic pain have become increasingly accepted within various comprehensive approaches, with rapid growth over recent decades [20]. Numerous CS-related biological mechanisms have been identified, including disruptions in both ascending and descending pathways of the central nervous system [21]. For patients with CS-induced pain, interventions targeting the brain and descending inhibitory pathways are more appropriate than peripheral-focused strategies [5]. Common clinical bottom–up approaches to CS include therapeutic exercise, manual therapy, pharmacological treatment, and surgery [22,23]. Additionally, Martarelli et al. [24] and Zou, Yeung, Quan, Boyden and Wang [11] reported that breathing interventions, particularly diaphragmatic breathing (also known as deep breathing or abdominal breathing), show potential in alleviating pain perception and emotional distress by promoting physiological equilibrium in fibromyalgia patients.

This aligns with reports indicating a correlation between chronic pain and inappropriate breathing patterns [25]. However, while the relationship between BPDs and chronic pain outcomes is frequently mentioned, direct correlational studies remain scarce. Therefore, this study aims to analyze the correlation between CS, a mechanistic basis for chronic pain, and BPDs, to provide additional considerations for chronic pain management.

## 2. Materials and Methods

### 2.1. Study Design

This study is a STROBE (Strengthening the Reporting of Observational Studies in Epidemiology)-compliant cross-sectional study designed to investigate the association between central sensitization and breathing pattern disorders.

### 2.2. Participants and Ethics

This study was approved by the Institutional Review Board (IRB) of Honam University (1041223-202405-HR-01). Study participants were church members who completed the Korean version of the Central Sensitization Inventory (CSI-K). The survey participants consisted of 190 middle-aged men and women over 40 years old, and evaluations were conducted for those who scored moderate or higher (≥40 points) on the survey. The study group was composed of volunteers who responded to recruitment notices, understood the study details and purpose, and expressed willingness to participate. The inclusion criteria were the following: adults in their 40s; CSI-K score of 40 or higher; those who agreed to the use and presentation of physical function assessment information and wished to participate in measurement experiments [15]. Exclusion criteria were the following: those with respiratory diseases; those with one or more infections or cancer; those who expressed intention to withdraw during the study period [26].

### 2.3. Sample Size

Based on previous research, the explanatory power (R^2^) of breathing pattern disorders and psychological factors was reported as 0.37, yielding an effect size (f^2^) of 0.58 for multiple regression analysis [27]. With a significance level (α) of 0.05, a statistical power (1 − β) of 0.90, and an anticipated inclusion of eight independent variables (general characteristics, central sensitization, respiratory mobility, respiratory muscle strength, breathing pattern, pain intensity, pain perception, and muscle tone and stiffness), a sample size of 40 participants was calculated to be required.

### 2.4. Outcomes

#### 2.4.1. Central Sensitization

The central sensitization inventory (CSI) is a self-reported questionnaire developed and validated to assess patients with CS [28,29]. In this study, the Korean version of the CSI (CSI-K) was used to evaluate the degree of CS among participants.

The CSI-K consists of two parts, A and B, but only Part A was used for scoring in this study. Part A is composed of 25 items, covering physical symptoms, emotional distress, headaches or jaw symptoms, and urological symptoms. To aid in the clinical interpretation of the CSI-K, five severity levels were established: asymptomatic (0–29 points), mild (30–39 points), moderate (40–49 points), severe (50–59 points), and extreme (60–100 points). A score of 40 or higher is considered the threshold for moderate or higher CS [13]. Therefore, in this study, the CSI-K was applicable to participants aged 15–69 years, and participants were selected based on having moderate or higher CS levels with a total score of 40 points or higher. The test–retest reliability (Intraclass Correlation Coefficient, ICC) of the CSI-K is 0.88, and its internal consistency (Cronbach’s α) is 0.94 [30].

#### 2.4.2. Respiratory Mobility

In this study, respiratory motion was measured using the manual assessment of respiratory motion (MARM). MARM evaluates the superior-lateral thoracic movement detected by the examiner’s hands on the posterolateral aspect of the thorax [31]. It is used to assess the relative contribution of upper and lower thoracic movement during five breathing instructions and postural conditions: 1. normal breathing in natural sitting, 2. sitting upright, 3. slow deep breathing, 4. breathing, and 5. normal breathing in natural sitting. The assessment begins with the participant seated on a backless chair or table. During the assessment, the examiner sits behind the participant, placing both hands on the lateral aspects of the rib cage with the entire hand comfortably positioned to avoid restricting respiratory motion. The thumbs are positioned parallel to the spine pointing vertically upward, with the other fingers extended horizontally. The fourth and fifth fingers should contact below the lower ribs to sense abdominal expansion.

The MARM results are calculated through angles measured from lines A and B drawn by the assessor, with the upper limit at 180° and the lower limit at 0°. Two variables are derived from the MARM assessment. The first variable is the angle between lines A and B, representing the degree or area of respiratory motion. This variable increases with deeper breathing and decreases with shallow breathing. The second variable is the average value of lines A and B, indicating breathing location. This variable increases with thoracic-dominant breathing and decreases with diaphragm-dominant breathing. A MARM area of less than 30° indicates abnormally shallow breathing, while a MARM average of greater than 100° indicates abnormally thoracic-dominant breathing [31]. The MARM Diagram is used for assessment documentation [31]. To ensure reliable assessment, all MARM evaluations throughout the study period were conducted by a single researcher. The test–retest reliability (ICC) of MARM is 0.85 [31].

#### 2.4.3. Respiratory Pattern

In this study, the Self-Evaluation of Breathing Questionnaire (SEBQ) was used to assess respiratory function. The SEBQ is a self-assessment questionnaire designed to measure symptoms and severity associated with breathing dysfunction [18]. It consists of 25 items scored on a 3-point scale, with individual items scored as 0 (never), 1 (occasionally), 2 (frequently), or 3 (very frequently), for a maximum total score of 75 points. Higher scores indicate greater degrees of breathing dysfunction [32]. In this study, the SEBQ was administered using translated individual items. While there is no established cutoff score for the SEBQ, following previous research, a score of 25 points was used as the threshold for breathing dysfunction [33]. The test–retest reliability (ICC) of the SEBQ is 0.89, and its internal consistency (Cronbach’s α) is 0.93 [14,34].

#### 2.4.4. Respiratory Strength

In this study, respiratory muscle strength was evaluated by measuring Maximal Inspiratory Pressure (MIP) and Maximal Expiratory Pressure (MEP) using a respiratory muscle strength measurement device (Pony FX MIP/MEP; Cosmed Inc., Rome, Italy). MIP represents the respiratory pressure generated during maximal inspiration following expiration, reflecting the strength of inspiratory muscles such as the diaphragm. MEP represents the respiratory pressure generated during maximal expiration following inspiration, reflecting the strength of expiratory muscles including the rectus abdominis, external oblique, internal oblique, and transversus abdominis [35]. The assessment was conducted in a seated position with the hip flexed at 90° [36].

To ensure accurate measurements, an experienced researcher provided thorough explanations and demonstrations to participants, ensuring complete understanding of the procedure before testing. Participants were instructed to wear a nose clip and firmly secure the mouthpiece to prevent air leakage. For maximal expiratory measurement, participants were instructed to “exhale maximally (maintain for 6 s),” during which they performed maximal oral expiration. Subsequently, for maximal inspiratory measurement, participants were instructed to “inhale maximally (maintain for 6 s),” during which they performed maximal oral inspiration. The MIP/MEP measurement test was performed three times for each participant, with the highest value used for analysis. The inter-rater reliability ICC range for the Pony FX MIP/MEP is 0.939–0.982 [37].

#### 2.4.5. Muscle Tone and Muscle Stiffness

The Myoton PRO (Myoton AS, Estonia and Myoton Ltd., London, UK) is a device that enables non-invasive and objective measurement of muscle mechanical properties [38]. It is also a highly valuable tool for biomechanical analysis of muscles that control body movement using various parameters including muscle contraction [39]. In this study, muscle tone and stiffness of the upper trapezius were measured in relation to CS and BPDs. Measurements were taken three times in a seated position, and the mean values were used for statistical analysis. The muscle tone meter was set to the multiscan mode with 5 repetitions per measurement, mechanical impulse tap time of 15 m/s, and interval of 0.8 s. The intra-rater reliability of the Myoton PRO ranges from 0.94 to 0.99 [40].

#### 2.4.6. Pain Intensity

The numeric pain rating scale (NPRS) is a unidimensional measurement tool for assessing pain intensity in patients with chronic pain [41,42]. In this study, the NPRS was used to measure pain severity. The NPRS consists of 11 points, with 0 representing no pain and 10 representing the worst possible pain [43]. Participants were asked to indicate the number corresponding to their pain intensity. Scores are interpreted as follows: 0 indicates no pain, 1–3 indicates mild pain, 4–6 indicates moderate pain, and 7–10 indicates severe pain [44]. The test–retest reliability (ICC) of the NPRS is 0.96 [41].

#### 2.4.7. Pain Cognition

The pain catastrophizing scale (PCS) was used to assess psychological aspects of pain. It is a 13-item self-assessment scale that measures catastrophizing in the context of actual or anticipated pain [45]. Each item is scored on a 5-point Likert-type scale including 0 (not at all), 1 (rarely), 2 (sometimes), 3 (frequently), and 4 (always), with total scores ranging from 0 to 52. Higher total scores indicate more negative pain perceptions and feelings of helplessness, reflecting higher levels of catastrophic thinking about pain [46].

This study utilized the Korean version of the Pain Catastrophizing Scale (K-PCS), which was modified and translated by Cho, et al. [47]. Following Sullivan et al.’s research, a threshold score of 20 was used to identify patients at risk for developing chronic pain. The K-PCS demonstrates a test–retest reliability (ICC) of 0.77 and internal consistency (Cronbach’s α) of 0.90 [47].

### 2.5. Data Analysis

All data in this study were analyzed using the statistical software SPSS version 25.0 (IBM, Armonk, NY, USA). The general characteristics of the participants were presented using descriptive statistics. The Shapiro–Wilk test was applied to assess the normality of the variables. Pearson’s correlation analysis was conducted to examine the linearity between variables. Simple linear regression analysis (multiple linear regression analysis) was used to verify the relationship of variance among variables with confirmed linearity. Prior to regression analysis, the following assumptions were assessed: (1) linearity through scatterplot examination, (2) independence of residuals using Durbin–Watson statistics, (3) homoscedasticity through residual plots, and (4) normality of residuals using the Shapiro–Wilk test. Multicollinearity was assessed using Variance Inflation Factor (VIF < 10). The level of statistical significance (α) was set at 0.05 for all analyses.

## 3. Results

**Figure 1:**
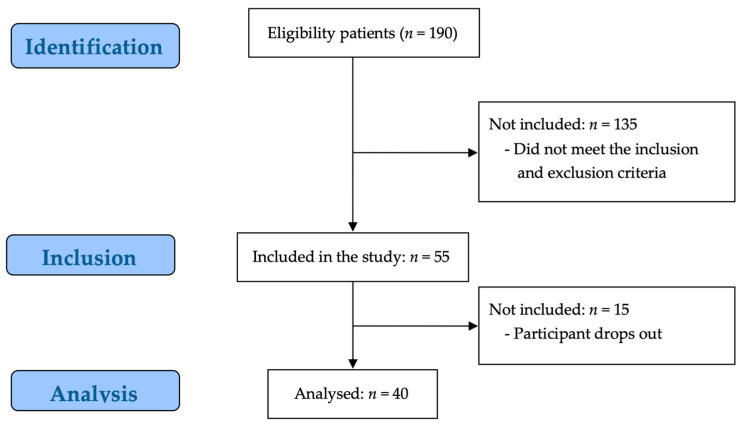
STROBE flow chart for recruitment.

Of the 190 potential participants, 135 participants were excluded by the evaluator for not meeting the study inclusion criteria, and 15 participants were excluded because they did not consent to participate in the study. Consequently, 40 participants were enrolled in the study (Figure 1).

### 3.1. Study Participant Characteristics

In this study, 40 participants with moderate or higher CS were enrolled, consisting of 7 males and 33 females. The general characteristics of the study participants were as follows: age 56.38 ± 8.05 years, height 160.75 ± 5.96 cm, weight 61.50 ± 9.00 kg, and BMI 23.80 ± 3.29 kg/m^2^ (Table 1).

### 3.2. Descriptive Analysis of Outcome Variables

Among participants with moderate to extreme CS, 82.5% (33 participants) showed breathing pattern disorders (MARM average ≥ 100°), and 42.5% (17 participants) reported severe pain intensity (NPRS usual pain ≥4). The mean CSI-K score was 51.30 ± 8.85, indicating moderate to severe central sensitization. Respiratory assessment revealed a mean MARM average of 111.15 ± 11.31°, exceeding the threshold for breathing pattern disorders. The mean MARM area was 42.35 ± 16.48°, indicating shallow breathing patterns. Respiratory muscle strength showed mean MIP of 70.08 ± 27.10 cm H_2_O and MEP of 75.23 ± 14.20 cm H_2_O. The SEBQ score averaged 23.48 ± 11.76, approaching the dysfunction threshold of 25 points. Pain measures showed mean usual pain of 3.15 ± 2.51 and worst pain of 5.08 ± 2.72 on the NPRS. The K-PCS score averaged 23.25 ± 12.65, indicating moderate pain catastrophizing. Muscle properties showed tone of 16.81 ± 2.25 Hz and stiffness of 340.45 ± 72.80 N/m.

### 3.3. Regression Analysis Between Variables

All regression models satisfied the assumptions of linearity, independence (Durbin–Watson ≈ 2.0), homoscedasticity, and normality of residuals (*p* > 0.05). No multicollinearity issues were detected (VIF < 3.0).

#### 3.3.1. Simple Regression Analysis

Simple regression analyses were performed on variables showing linear relationships among participants with CSI-K scores of 40 or higher, with results presented in Table 2.

Among respiratory variables, the regression model between MARM average and MARM area yielded statistical significance (F = 23.389, *p* < 0.05), with an explained variance of 36.3%. MARM average exhibited a significant positive influence on MARM area (β = 0.603, *p* < 0.05). The regression model between MIP and MEP demonstrated statistical significance (F = 25.421, *p* < 0.05), accounting for 63.3% of the variance, with a significant positive relationship (β = 0.633, *p* < 0.05). MEP showed a significant negative relationship with SEBQ (F = 7.058, *p* < 0.05, R^2^ = 0.157, β = −0.396).

Regarding pain variables, the regression model between NPRS usual pain and worst pain was statistically significant (F = 55.491, *p* < 0.05), explaining 59.4% of the variance with a strong positive relationship (β = 0.770, *p* < 0.05). K-PCS demonstrated significant positive relationships with both NPRS usual pain (F = 12.729, *p* < 0.05, R^2^ = 0.237, β = 0.487) and worst pain (F = 6.324, *p* < 0.05, R^2^ = 0.143, β = 0.378).

**Figure 2:**
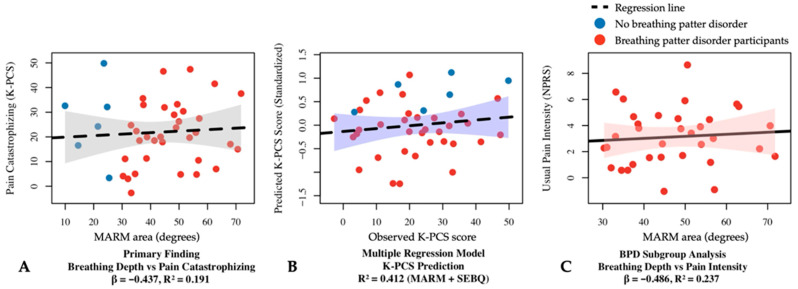
Regression analysis results between respiratory and pain variables in participants with central sensitization. (**A**) Simple linear regression showing the relationship between MARM area and pain catastrophizing (β = −0.437, R^2^ = 0.191, *p* < 0.05). (**B**) Multiple linear regression model for K-PCS prediction using MARM area and SEBQ as predictors (R^2^ = 0.412, *p* < 0.05). (**C**) Simple linear regression in the breathing pattern disorders subgroup (MARM average ≥ 100°) showing the relationship between MARM area and usual pain intensity (β = −0.486, R^2^ = 0.237, *p* < 0.05). BPD, breathing pattern disorders; K-PCS, Korean version of pain catastrophizing scale; MARM, manual assessment of respiratory motion; NPRS, numeric pain rating scale; SEBQ, self-evaluation of breathing questionnaire.

Examining respiratory–pain relationships, a significant negative correlation was found between K-PCS and MARM area (F = 8.957, *p* < 0.05, R^2^ = 0.191, β = −0.437 (Figure 2A). The relationship between K-PCS and SEBQ yielded a significant regression model (F = 14.699, *p* < 0.05, R^2^ = 0.279) with a significant positive relationship (β = 0.528, *p* < 0.05).

The regression model between Muscle Tone and Muscle Stiffness demonstrated the highest explained variance (87.5%) (F = 264.823, *p* < 0.05), exhibiting a strong positive relationship (β = 0.935, *p* < 0.05).

#### 3.3.2. Sub-Analysis: Breathing Pattern Disorders Group

In participants with breathing pattern disorders, stronger relationships emerged between respiratory mechanics and pain measures. MARM area demonstrated significant negative relationships with pain intensity: NPRS usual pain (F = 9.067, *p* < 0.05, R^2^ = 0.237, β = −0.486) (Figure 2C) and worst pain (F = 6.773, *p* < 0.05, R^2^ = 0.179, β = −0.423). The relationship with K-PCS was particularly strong (F = 17.857, *p* < 0.05, R^2^ = 0.366, β = −0.605). K-PCS also showed a significant positive relationship with SEBQ (F = 17.024, *p* < 0.05, R^2^ = 0.354, β = 0.595) and a negative relationship with MEP (F = 5.402, *p* < 0.05, R^2^ = 0.148, β = −0.385).

The relationship between muscle tone and muscle stiffness exhibited notably high explained variance (94.1%) with a strong positive relationship (F = 493.335, *p* < 0.05, β = 0.970). A significant negative correlation was observed between MARM average and muscle stiffness (F = 4.317, *p* < 0.05, R^2^ = 0.127, β = −0.356).

#### 3.3.3. Sub-Analysis: Severe Pain Group

In participants with severe pain intensity, central sensitization showed stronger relationships with multiple variables. CSI-K demonstrated significant positive relationships with SEBQ (F = 16.096, *p* < 0.05, R^2^ = 0.518, β = 0.719), K-PCS (F = 12.030, *p* < 0.05, R^2^ = 0.445, β = 0.667), and muscle stiffness (F = 6.361, *p* < 0.05, R^2^ = 0.298, β = 0.546).

Among respiratory variables, significant relationships were found between MARM area and MEP (F = 6.220, *p* < 0.05, R^2^ = 0.246, β = 0.541), and between MIP and SEBQ (F = 5.402, *p* < 0.05, R^2^ = 0.265, β = −0.515). K-PCS showed a negative relationship with MEP (F = 7.153, *p* < 0.05, R^2^ = 0.308, β = −0.555).

Muscle tone and muscle stiffness maintained a strong positive relationship (F = 215.724, *p* < 0.05, R^2^ = 0.935, β = 0.967). K-PCS demonstrated a significant positive relationship with muscle stiffness (F = 8.199, *p* < 0.05, R^2^ = 0.353, β = 0.594).

#### 3.3.4. Multiple Regression Analysis

Multiple regression analyses were performed to examine the combined effects of variables, with results presented in Table 3.

For MARM area prediction in the total group, the regression model with MARM average and K-PCS yielded statistical significance (F = 20.428, *p* < 0.05), explaining 52.5% of the variance (R^2^ = 0.525, adj R^2^ = 0.499). MARM average showed a significant positive influence (β = 0.578, *p* < 0.05), while K-PCS demonstrated a significant negative influence (β = −0.415, *p* < 0.05).

Multiple regression analysis for K-PCS prediction with MARM area and SEBQ was statistically significant (F = 12.980, *p* < 0.05), explaining 41.2% of the variance (R^2^ = 0.412, adj R^2^ = 0.381) (Figure 2B). MARM area exhibited a significant negative influence (β = −0.369, *p* < 0.05), while SEBQ showed a significant positive influence (β = 0.476, *p* < 0.05).

In the breathing pattern disorders subgroup, NPRS usual pain prediction using NPRS worst pain (β = 0.647, *p* < 0.05) and K-PCS (β = 0.288, *p* < 0.05) explained 66.4% of variance (F = 29.624, *p* < 0.05). K-PCS prediction using MARM area (β = −0.476, *p* < 0.05) and SEBQ (β = 0.463, *p* < 0.05) explained 56.4% of variance (F = 19.388, *p* < 0.05).

In the severe pain subgroup, MARM area prediction using MARM average (β = 0.513, *p* < 0.05) and MEP (β = 0.531, *p* < 0.05) explained 55.6% of variance (F = 8.756, *p* < 0.05).

The most comprehensive model examined muscle stiffness prediction using CSI-K, K-PCS, and muscle tone, which demonstrated statistical significance (F = 195.627, *p* < 0.05) with remarkably high explained variance of 97.8% (R^2^ = 0.978, adj R^2^ = 0.973). All three variables showed significant positive influences: CSI-K (β = 0.124, *p* < 0.05), K-PCS (β = 0.132, *p* < 0.05), and muscle tone (β = 0.861, *p* < 0.05).

All regression analyses satisfied the assumptions of residual independence and showed no multicollinearity issues (VIF < 10).

## 4. Discussion

This study specifically aimed to investigate the relationship between breathing pattern disorders and pain in individuals with clinically significant central sensitization (CSI-K ≥ 40). The cut-off score of 40 was chosen based on validated clinical significance thresholds [13], as our research focused on examining these relationships in populations with established moderate to severe central sensitization.

The regression analyses revealed significant interrelationships among respiratory function, pain perception, and psychological factors in participants with moderate or higher CS. While direct relationships between the degree of central sensitization and breathing pattern disorders were not consistently demonstrated across all participants, 82.5% (33 participants) had breathing pattern disorders, and 42.5% (17 participants) had severe pain intensity. The regression models provided quantitative evidence for these complex relationships.

Among respiratory variables, linear relationships were identified where MARM average positively influenced MARM area, with the regression equation (MARM area) = 49.064 + 0.314(MARM average) explaining 36.3% of the variance. A strong relationship was discovered where MEP positively influenced MIP, with the regression equation (MIP) = −20.838 + 1.209 (MEP) explaining 63.3% of the variance. These results indicate that breathing depth, MIP, and MEP interact with and influence each other through complex neurophysiological mechanisms in the central nervous system. When the nervous system becomes hypersensitive due to CS, abnormal breathing patterns are reinforced through altered respiratory center sensitivity in the brainstem, resulting in shallower breathing depth and unstable pressure control [48,49]. The altered neural control affects both inspiratory and expiratory muscle function by changing motor neuron recruitment patterns and muscle fiber activation thresholds. Additionally, this neurophysiological interaction helps explain why expiratory muscle weakness is closely related to respiratory function problems, as CS causes abnormal respiratory muscle control and muscle weakness, resulting in reduced efficiency of the expiration process [50]. The negative relationship between MEP and SEBQ (β = −0.396, *p* < 0.05) provides potential clinical indicators for assessing respiratory dysfunction and monitoring treatment progress in CS patients.

Regarding pain variables, linear relationships were found where K-PCS positively influenced both usual pain (β = 0.487, *p* < 0.05) and worst pain (β = 0.378, *p* < 0.05). The strong relationship between usual and worst pain (β = 0.770, *p* < 0.05, R^2^ = 0.594) demonstrates the consistency of pain experience in CS patients. These results are explained by higher pain levels correlating with stronger negative pain perception, which appears to be because psychological factors trigger and weaken central nervous system and autonomic nervous system sensitization [51]. Negative pain perception strengthens pain pathways through multiple levels of neuroplastic adaptation in the nervous system. This adaptation process involves enhanced synaptic efficiency in pain pathways, altered descending pain modulation, and autonomic nervous system dysregulation. The relationship between pain perception and central sensitization creates a self-perpetuating cycle where breathing pattern disorders contribute to sympathetic hyperarousal, which in turn maintains central sensitization [52]. This study provides quantitative demonstration of this relationship in CS patients, suggesting that interventions targeting breathing pattern modification could serve as a potential therapeutic approach.

The most significant finding was the relationship between respiratory mechanics and pain cognition. The regression equation (K-PCS) = 15.522–0.369(MARM area) + 0.476(SEBQ) explained 41.2% of the variance in pain catastrophizing. The negative relationship between MARM area and K-PCS (β = −0.437 in simple regression, β = −0.369 in multiple regression) provides the first quantitative evidence of how breathing mechanics directly influence pain cognition in CS patients. This relationship appears to be mediated through several interconnected pathways, including direct effects of mechanical breathing patterns on interoceptive processing, autonomic nervous system modulation through respiratory rhythm, and psychological impacts of breathing patterns on anxiety and stress responses [53,54]. The regression equation’s substantial explanatory power suggests clinical significance for therapeutic interventions targeting breathing patterns in CS patients. This finding is particularly important as it demonstrates how rapid shallow breathing patterns cause psychological adverse effects such as depression and anxiety along with sympathetic hyperactivation, leading to negative pain cognition.

The subgroup analyses revealed stronger and more specific relationships. In participants with breathing pattern disorders (MARM average ≥ 100°), MARM area demonstrated significant negative relationships with pain intensity: usual pain (β = −0.486, *p* < 0.05, R^2^ = 0.237) and worst pain (β = −0.423, *p* < 0.05, R^2^ = 0.179). The relationship with pain catastrophizing was particularly strong (β = −0.605, *p* < 0.05, R^2^ = 0.366). These findings indicate that shallow breathing patterns induce and exacerbate widespread pain centered around the posterior neck, and that chronic pain can be controlled through slow deep breathing interventions [2,55]. The derived regression equations provide clinical tools for predicting pain levels based on breathing patterns and support the integration of breathing assessment into standard CS patient evaluation protocols. This relationship suggests that breathing pattern modification could be considered a primary therapeutic target rather than just a secondary intervention.

In the severe pain subgroup (NPRS ≥ 4), central sensitization showed stronger relationships with multiple variables. CSI-K demonstrated significant positive relationships with SEBQ (β = 0.719, *p* < 0.05, R^2^ = 0.518), K-PCS (β = 0.667, *p* < 0.05, R^2^ = 0.445), and muscle stiffness (β = 0.546, *p* < 0.05, R^2^ = 0.298). These results suggest that CS intensification is closely related to respiratory dysfunction, negative pain perception, and muscle tension [56,57,58]. This results in a vicious cycle where neural hypersensitization, autonomic nervous system imbalance, psychological stress, and pain interact complexly, leading to stronger pain perception, irregular breathing, muscle stiffness, and reinforced negative pain perception [59,60,61].

The most comprehensive model examined muscle stiffness prediction, which demonstrated remarkably high explained variance of 97.8% using CSI-K (β = 0.124), K-PCS (β = 0.132), and muscle tone (β = 0.861). This represents a significant advancement in understanding the complex relationship between muscle properties and central sensitization. The derived regression equation provides a potential clinical tool for predicting CS progression based on muscle parameters and monitoring treatment effectiveness. The strong correlation between muscle tone and stiffness (r = 0.970) suggests a potential new biomarker for CS severity, though age-related factors need to be carefully considered in clinical interpretation. In this study, correlations between upper trapezius (a representative accessory respiratory muscle) tension and stiffness and respiratory variables could not be confirmed. However, participants’ mean upper trapezius muscle tone value of 16.81 ± 2.25 Hz and stiffness value of 340.45 ± 72.80 N/m were similar to the average values for women in their 50s (tone: 17.76 ± 1.19 Hz, stiffness: 342.45 ± 14.34 N/m), suggesting these results are due to participants’ average age rather than CS or BPDs [62].

Consequently, the strengths and limitations of this study are as follows. As a strength, while direct relationships between CS level and BPDs were not consistently demonstrated, significant relationships between respiratory and pain variables were identified through regression analysis in individuals with moderate or higher CS. The selection criteria (CSI-K ≥ 40) represent purposive sampling rather than selection bias, as our research question specifically targeted individuals with clinically significant central sensitization. This focused approach allows for more detailed examination of breathing patterns and pain relationships in this specific clinical population.

However, this study had the following limitations. First, participant recruitment was limited to specific locations, resulting in limitations in general characteristics. Second, 80% of participants were aged 50 or above, allowing age-related changes to influence outcome variables. Lastly, the accessory respiratory muscle was limited to only the upper trapezius, and muscle tone and stiffness showed a high positive correlation (r = 0.970). Since this high correlation between muscle tone and stiffness could indicate underlying muscle weakness rather than active muscle tension, relationship confirmation might be possible through pain pressure threshold measurement using an algometer.

Therefore, future studies should consider these limitations by recruiting CS participants from diverse groups and age ranges for better generalization, and quantitative analysis of various accessory respiratory muscles’ activation levels rather than a single muscle is deemed necessary. Future research directions should specifically focus on (1) longitudinal studies to establish causality between breathing patterns and CS progression; (2) intervention studies targeting breathing pattern modification as a primary treatment approach; (3) development of standardized protocols for breathing assessment in CS patients; (4) exploration of the relationship between breathing patterns and other autonomic markers; (5) investigation of age-specific breathing pattern characteristics in CS patients; (6) investigation of these relationships across the full spectrum of CS severity, including mild cases, to provide complementary insights.

Additionally, studies examining the effectiveness of breathing interventions in modifying pain perception and central sensitization would provide valuable clinical evidence. These research directions would contribute to establishing breathing pattern assessment and modification as core components of CS management protocols, potentially leading to more effective treatment strategies for patients with central sensitization.

## 5. Conclusions

In conclusion, this study demonstrates significant interrelationships among respiratory function, pain perception, and psychological factors in individuals with moderate to severe central sensitization through comprehensive regression analysis. The high prevalence of breathing pattern disorders (82.5%) and severe pain intensity (42.5%) in CS patients suggests that respiratory dysfunction is an integral component of the pain experience. The strong regression relationships between breathing mechanics (MARM area) and pain measures, along with the remarkably high explanatory power (97.8%) of the muscle properties regression model, provide quantitative evidence for these relationships. These findings highlight the importance of incorporating breathing pattern assessment in the clinical management of CS patients, while the identified regression equations offer practical tools for patient evaluation. Furthermore, the demonstrated relationships between breathing patterns and pain catastrophizing suggest that breathing pattern modification could serve as a primary therapeutic target, potentially opening new avenues for more effective CS management strategies.

## Figures and Tables

**Table 1:** General characteristics of the participants.

Variables	Mean ± SD
Sex (male/female)	7/33
Age (years)	56.38 ± 8.05
Height (cm)	160.75 ± 5.96
Weight (kg)	61.50 ± 9.00
BMI (kg/m^2^)	23.80 ± 3.29

BMI, body mass index; SD: standard deviation.

**Table 2:** The simple regression analysis between variables.

Dependent Variable	Independent Variable	Unstandardized Coefficients		Standardized Coefficients	t (*p*)	F	R^2^
		B	SE	β			
Simple regression analysis between respiratory variables: CSI-K (≥40 points) (*n* = 40)							
MARM; area	(constant)	49.064	4.817		10.186 (0.000)	8.957 **	0.191
	K-PCS	−0.686	0.229	−0.437	−2.992 (0.005)		
MIP	(constant)	−20.838	18.342		−1.136 (0.263)	25.421 **	0.633
	MEP	1.209	0.240	0.633	5.042 (0.000)		
MEP	(constant)	83.188	3.653		22.772 (0.000)	7.058 *	0.157
	SEBQ	−0.407	0.153	−0.396	−2.657 (0.011)		
Simple regression analysis between pain variables: CSI-K (≥40 points) (*n* = 40)							
NPRS; usual pain	(constant)	−1.161	0.604		−1.922 (0.062)	55.491 **	0.594
	NPRS; worst pain	0.824	0.111	0.770	7.449 (0.000)		
NPRS; usual pain	(constant)	2.182	0.380		5.741 (0.000)	12.729 **	0.237
	K-PCS	0.061	0.017	0.487	3.568 (0.004)		
NPRS; worst pain	(constant)	4.472	0.382		11.702 (0.000)	6.324 **	0.143
	K-PCS	0.046	0.018	0.378	2.515 (0.016)		
Simple regression analysis between other variables: CSI-K (≥40 points) (*n* = 40)							
MARM; area	(constant)	49.064	4.817		10.186 (0.000)	8.957 **	0.191
	K-PCS	−0.686	0.229	−0.437	−2.992 (0.005)		
SEBQ	(constant)	11.219	2.878		3.899 (0.000)	14.699 **	0.279
	K-PCS	0.525	0.137	0.528	3.834 (0.000)		
Muscle Tone	(constant)	6.967	0.618		11.271 (0.000)	264.823 **	0.875
	Muscle Stiffness	0.029	0.002	0.935	16.273 (0.000)		
Sub-analysis; simple regression analysis between variables: MARM; average (≥100 degrees) (*n* = 33)							
MARM; area	(constant)	59.948	5.977		10.030 (0.000)	9.607 **	0.237
	NPRS; usual pain	−5.056	1.631	−0.486	−3.099 (0.004)		
	(constant)	68.348	9.898		6.905 (0.000)	6.773 *	0.179
	NPRS; worst pain	−4.618	1.774	−0.423	−2.603 (0.014)		
	(constant)	56.852	4.093		13.891 (0.000)	17.857 **	0.366
	K-PCS	−0.806	0.191	−0.605	−4.226 (0.000)		
MEP	(constant)	81.968	3.627		22.600 (0.000)	5.402 **	0.148
	K-PCS	−0.393	0.169	−0.385	−2.324 (0.027)		
SEBQ	(constant)	10.599	3.160		3.354 (0.002)	17.024 **	0.354
	K-PCS	0.608	0.147	0.595	4.126 (0.000)		
Muscle Tone	(constant)	6.418	0.480		13.362 (0.000)	493.335 **	0.941
	Muscle Stiffness	0.031	0.001	0.970	22.211 (0.000)		
Muscle Stiffness	(constant)	707.909	174.285		4.062 (0.000)	17.024 **	0.354
	MARM; average	−3.206	1.513	−0.356	−2.119 (0.042)		
Sub-analysis; Simple regression analysis between respiratory variables: NPRS; usual pain (≥4 points) (*n* = 17)							
MARM; area	(constant)	−9.989	17.290		−0.578 (0.572)	6.220 **	0.246
	MEP	0.572	0.229	0.541	2.494 (0.025)		
MIP	(constant)	84.648	8.850		9.565 (0.000)	5.402 *	0.265
	SEBQ	−0.691	0.297	−0.515	−2.324(0.035)		
Sub-analysis; Simple regression analysis between other variables: NPRS; usual pain (≥4 points) (*n* = 17)							
Muscle Tone	(constant)	6.629	0.681		9.739 (0.000)	215.724 **	0.935
	Muscle Stiffness	0.030	0.002	0.967	3.242 (0.000)		
Muscle Stiffness	(constant)	268.996	24.868		10.817 (0.000)	8.199 *	0.353
	K-PCS	2.616	0.914	0.594	2.863 (0.012)		
CSI-K	(constant)	42.098	2.020		20.837 (0.000)	16.096 **	0.518
	SEBQ	0.272	0.068	0.719	4.012 (0.001)		
	(constant)	41.780	2.353		17.753 (0.000)	12.030 **	0.445
	K-PCS	0.300	0.086	0.667	3.468 (0.003)		
	(constant)	30.283	7.404		4.090 (0.001)	6.361 *	0.298
	Muscle Stiffness	0.056	0.022	0.546	2.522 (0.023)		

CSI-K, central sensitization inventory—Korean version; K-PCS, Korean pain catastrophizing scale; MARM, manual assessment of respiratory motion; MEP, maximal expiratory pressure; MIP, maximal inspiratory pressure; NPRS, numeric pain rating scale; SEBQ, self-evaluation of breathing questionnaire. * *p* < 0.05, ** *p* < 0.01.

**Table 3:** The multiple regression analysis between variables.

Dependent Variable	Independent Variable	Unstandardized Coefficients		Standardized Coefficients	t (*p*)	F	R^2^
		B	SE	β			
Multiple regression analysis between variables: CSI-K (≥40 points) (*n* = 40)							
MARM; area	(constant)	−75.530	24.717		−3.056 (0.004)	20.428 *	0.525
	MARM; average	1.116	0.219	0.578	5.100 (0.000)		
	K-PCS	−0.651	0.178	−0.415	−3.658 (0.001)		
K-PCS	(constant)	15.522	24.717		3.381 (0.002)	20.428 *	0.525
	MARM; area	−0.235	0.081	−0.369	−2.898 (0.006)		
	SEBQ	0.479	0.128	0.476	3.735 (0.001)		
Sub-analysis; Multiple regression analysis between variables: MARM; average (≥100 degrees) (*n* = 33)							
NPRS; usual pain	(constant)	−1.012	0.621		−1.629 (0.114)	29.624 *	0.664
	NPRS; worst pain	0.678	0.123	0.647	5.498 (0.000)		
	K-PCS	0.037	0.015	0.288	2.452 (0.020)		
K-PCS	(constant)	22.527	5.657		3.982 (0.000)	19.388 *	0.564
	MARM; area	−0.357	0.094	−0.476	−3.794 (0.001)		
	SEBQ	0.454	0.123	0.463	3.693 (0.001)		
Sub-analysis; Multiple regression analysis between variables: NPRS; usual pain (≥4 points) (*n* = 17)							
MARM; area	(constant)	−95.780	33.026		−2.900 (0.012)	8.756 *	0.556
	MARM; average	0.779	0.271	0.513	2.877 (0.012)		
	MEP	0.560	0.188	0.531	2.978 (0.010)		
Muscle Stiffness	(constant)	−201.736	29.960		−6.734 (0.000)	195.627 *	0.978
	CSI-K	1.210	0.542	0.124	2.233 (0.044)		
	K-PCS	0.583	0.250	0.132	2.329 (0.037)		
	Muscle Tone	27.883	1.489	0.861	18.731 (0.000)		

CSI-K, central sensitization inventory—Korean version; K-PCS, Korean pain catastrophizing scale; MARM, manual assessment of respiratory motion; MEP, maximal expiratory pressure; NPRS, numeric pain rating scale; SEBQ, self-evaluation of breathing questionnaire. * *p* < 0.05.

## Data Availability

The data presented in this study are available on request from the corresponding author.
